# Evaluating the Performance and Perception of a Stoma Bag Full-Circle Filter in People with a Colostomy or an Ileostomy—Two Randomized Crossover Trials

**DOI:** 10.3390/healthcare11030369

**Published:** 2023-01-28

**Authors:** Tracey Virgin-Elliston, Pernille Nonboe, Esben Bo Boisen, Henrik Koblauch

**Affiliations:** 1 Department of Colorectal Surgery, Chelsea and Westminster NHS Foundation Trust, London SW10 9NH, UK; 2 Coloplast A/S, Holtedam 1-3, DK-3050 Humlebæk, Denmark

**Keywords:** stoma, stoma bag, colostomy, ileostomy, ostomy, ballooning, randomized controlled trial, crossover trial, filter, Sensura Mio

## Abstract

Stoma bag filter-related issues, such as ballooning (the bag filling with gas), remain highly prevalent among users. The full-circle filter was purposely designed to reduce ballooning through the inclusion of a unique, full-circle pre-filter. Two similar randomized crossover trials were conducted to compare the performance of the full-circle filter with a dual filter in adults with a colostomy (n = 20) or an ileostomy (n = 20). The frequency of ballooning was significantly lower with the full-circle filter versus the dual filter in participants with a colostomy (*p* < 0.0007) and in participants with an ileostomy (*p* < 0.0001). No significant differences were observed in the frequency of other issues (pancaking, odor problems, and ostomy solution discretion) between the filters. On average, participants with an ileostomy wore ostomy solutions with the full-circle filter for 3.3 h longer than ostomy solutions with the dual filter (*p* < 0.0001); wear-time in users with a colostomy was comparable between the filters. Considering the lack of published research on stoma bag filters and the high prevalence of filter-related issues, these data provide important information for health care practitioners who support people living with a stoma.

## 1. Introduction

Ostomy surgery can be a necessary and, potentially, life-saving procedure for patients with various gastrointestinal or urological conditions and traumas [1,2,3]. Although international estimates are unavailable, a 2012 report suggested that 700,000 people in Europe were living with a stoma [1]. In the US, estimates of the number of people living with a stoma range from 750,000 to 1 million, and approximately 100,000 new stoma surgeries are performed annually [4].

An ostomy procedure diverts the intestinal or urinary tract through a surgical opening in the abdomen (a stoma), leaving many individuals incontinent and, therefore, reliant on a stoma bag to collect the stomal effluent [1,2]. The availability of well-fitting and functional ostomy product solutions is crucial for the health, well-being, and general health-related quality of life of people living with a stoma [1,5,6,7]. Although considerable advances have been made in the design of ostomy solutions, studies show that issues such as ballooning (the bag filling with gas), pancaking (effluent collecting around the stoma when there is no air/space in the bag), leakage of stomal effluent, and odor problems remain highly prevalent among users and can interfere with multiple aspects of daily life [8,9,10,11,12]. Indeed, people living with a stoma have highlighted that addressing problems with ostomy solutions should be a research priority [13].

Most modern stoma bags include a filter to allow gas to escape from the bag [4,14]. Active charcoal is incorporated in most filters to absorb odors from the escaping gas [4]. However, if the filter cannot release enough gas from the bag, or it becomes wet or blocked due to effluent from the stoma, ballooning may occur [14]. Ballooning is a common filter-related issue that can lead to a cascade of additional problems [9,14]. In an online survey of >4000 people living with a stoma, 91% had experienced ballooning [9]. Because of ballooning, 39% of respondents worried about leakage of stomal effluent, and one in three were concerned that others would notice the profile of the stoma bag and/or its odor [9]. A further 46% of people living with a stoma reported sleep disruption due to ballooning [9]. However, despite the high prevalence of filter-related issues [9,10], there is a lack of published research evaluating the performance of stoma bag filters, which can make it difficult for health care practitioners to make evidence-based decisions when faced with filter-related issues.

A full-circle filter was purposely designed to reduce the occurrence of ballooning and its related issues. The filter system includes a unique, full-circle foam pre-filter, which allows gas to exit the bag, whilst protecting the filter from stomal effluent. Although other filter systems, such as the dual filter, also include pre-filters to protect the filter from contamination, the larger size of the full-circle pre-filter provides greater protection from contamination with stomal effluent, potentially prolonging the time to filter dysfunction. Therefore, it was hypothesized that use of the full-circle filter would reduce the occurrence of ballooning events compared with the existing filters (single and dual). To investigate this hypothesis, two randomized crossover trials were conducted in Denmark. These trials aimed to evaluate the performance and perception of the full-circle filter in people living with a colostomy or an ileostomy.

## 2. Materials and Methods

### 2.1. Trial Design

**Figure 1:**
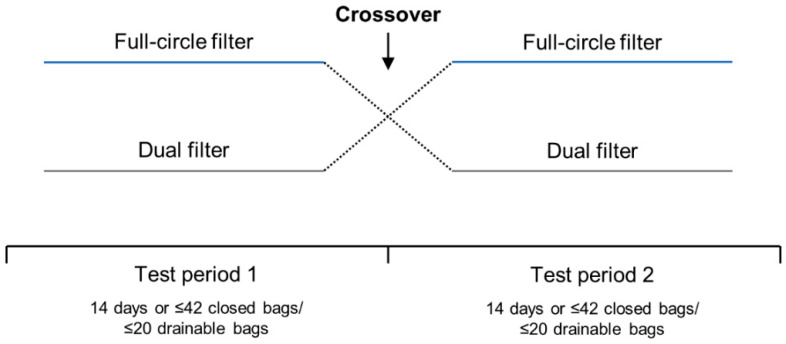
Two-way crossover study design used for the two trials.

Two 4-week, open-label, randomized, two-way crossover trials were conducted between January and May 2011 to compare the performance and perception of the full-circle filter with the dual filter in people with a colostomy (CP210OC; ClinicalTrials.gov identifier: NCT01273038) or an ileostomy (CP211OC; ClinicalTrials.gov identifier: NCT01272869). The data manager generated a randomization list prior to trial initiation using the randomization software, Med Stat version 2.1. Upon enrollment, participants were randomized 1:1 to use one-piece ostomy solutions with the full-circle filter or one-piece ostomy solutions with the dual filter in Test Period 1 (Figure 1). Identical sealed randomization envelopes were provided, and randomization numbers were recorded on the participant questionnaires.

In each test period, participants used bags with one type of filter for 14 days or a maximum of 42 closed (participants with a colostomy) or 20 drainable (participants with an ileostomy) bags. Participants were instructed to change their ostomy solution as frequently as they usually would, or whenever they considered it necessary (including when ballooning occurred). If a participant used >42 closed or >20 drainable bags in <14 days, they were required to stop the test period early but were not excluded from the trial. At the crossover point, participants were instructed to continue using the current ostomy solution until a change was necessary. All participants were contacted by telephone at the crossover point (14 days) to ensure their well-being, to check that they were following the protocol, to clarify any issues with the ostomy solution, and to remind them to cross over. The trials did not include a washout period as it was assumed that any carryover effect would be negligible due to all ostomy solutions having the same type of adhesive and pouch. Furthermore, as the outcomes of the trials were related to device performance rather than direct biological or behavioral changes (e.g., skin issues or quality of life), a washout period was not considered necessary.

During the trials, participants were required to fill out a questionnaire each time they changed their ostomy solution. The questionnaire, devised specifically for the trials, included items assessing the time of change; reason for change (e.g., ballooning); problems with ballooning, pancaking, and odor; and perception of ostomy solution discretion. Individuals who provided answers that required further clarification were invited to an optional interview. The trials ended when all questionnaires had been returned and all interviews had been conducted. After trial termination, all participants who experienced adverse events (AEs) related to the trial devices (ADEs) were followed until these events resolved (if possible, due to practical circumstances). Follow-up of ADEs also applied to participants who discontinued the trials early.

### 2.2. Participants

Following approval from the Danish National Committee on Biomedical Research Ethics and the Danish Medicines Agency, potential participants were identified from a database used by Coloplast A/S (permission from the Data Protection Agency 2009-42-1399) using the following criteria: have a colostomy or an ileostomy, use an ostomy solution with a flat baseplate, and do not irrigate their stoma. Letters of inquiry were sent to individuals who met the criteria and had consented to receive details of clinical trials; those who responded positively were invited to an in-person meeting with the investigator or one of their representatives. All participants provided written informed consent prior to enrollment.

Adults aged ≥18 years capable of completing the participant questionnaire and who had a colostomy or an ileostomy for ≥6 months, experienced ≥1 ballooning event per week, were able to manage the application and removal of ostomy solutions without assistance, were able to use an ostomy solution with a flat baseplate, and had a stoma with a diameter <60 mm were eligible for inclusion in the trials. People who irrigated their stoma, had bleeding or broken skin around the stoma, were currently receiving or had received (in the past 2 months) radiotherapy and/or chemotherapy, who were pregnant or breastfeeding, or were currently participating in another clinical study were excluded.

The trials were conducted in accordance with the Declaration of Helsinki, the European Medical Device Directive (2007/47/EC), ISO 14155 (Parts 1 and 2), ISO 14971, and ISO 10993-1 (E).

### 2.3. Filters and Ostomy Solutions

**Figure 2:**
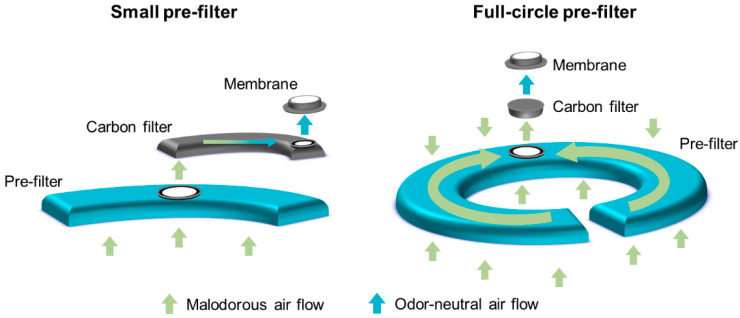
Schematic of the small pre-filter (used in the dual filter system) and the full-circle pre-filter.

The full-circle and dual filters (Figure 2) were mounted in identical non-sterile, one-piece, midi-size bags with flat custom-cut (66 mm maximum) baseplates (manufactured by Coloplast A/S, Humlebæk, Denmark). The baseplates had an identical hydrocolloid adhesive skin barrier and were mounted on an opaque stoma bag. At the time of the study, stoma bags with the full-circle filter were not commercially available and were non-CE marked, whilst bags with the dual filter were commercially available and CE-marked. Due to differences in the consistency of colostomy and ileostomy effluent, two variants of the full-circle filter were developed. Closed bags (for colostomies) include a foam pre-filter with a more open structure than the pre-filter in drainable bags (for ileostomies).

### 2.4. Outcomes

The primary endpoint was the difference in the frequency of ballooning between ostomy solutions with the full-circle filter and with the dual filter. Frequency of ballooning was calculated as the number of stoma bags that ballooned (according to the participant questionnaire) divided by the total number of bags used. Further assessments of ballooning frequency (all determined using the participant questionnaire) included the number of ballooning events (bags that ballooned) per person, the number of ballooning events per person per day, the number of ballooning events per bag (for each participant), and the distribution of ballooning events over 24 h.

Secondary endpoints comprised the difference in time to ballooning with the full-circle filter versus the dual filter, frequency of pancaking (number of bags that pancaked divided by the total number of bags), and frequency of odor problems (number of bags with odor problems divided by the total number of bags), which were all assessed using the participant questionnaire. Ostomy solution discretion with the full-circle filter versus the dual filter was assessed by an item in the participant questionnaire (‘how bothered were you by the fact that the product could be seen under your clothing?’), rated on a 5-point scale (from not at all to very much).

The difference in ostomy solution wear-time with the full-circle filter versus the dual filter was assessed in an additional analysis. Safety was assessed by recording AEs, ADEs, serious AEs, and serious ADEs.

### 2.5. Statistical Analysis

#### 2.5.1. Sample Size

Sample size calculations were based on the detection of a significant difference on the primary endpoint (frequency of ballooning) between the full-circle filter and the dual filter. From the findings of a similar study previously conducted by Coloplast A/S (CP201OC), it was assumed that ballooning would occur at a rate of 0.34 events per day with the dual filter; the full-circle filter was estimated to reduce the rate to 0.17 events per day. Based on an observation time of 14 days per filter, a significance level of 0.05, a power of approximately 80% and, to account for a dropout rate of 20%, a sample size of 20 was chosen for each trial.

#### 2.5.2. Analysis Sets and Outcomes

Outcomes were analyzed separately for each trial (i.e., for participants with a colostomy and for participants with an ileostomy); no statistical comparisons were performed between these two groups. Baseline demographics and clinical characteristics for each trial population are reported using descriptive statistics.

All outcomes were analyzed for the intention-to-treat (ITT) analysis set, which comprised people who were enrolled in the trial and who tested at least one study product. As a sensitivity analysis, the primary endpoint was also analyzed in the per-protocol (PP) analysis set. The PP analysis set comprised people in the ITT analysis set who completed the trial in compliance with the protocol, tested bags with each type of filter in consecutive order without using their own products, and used each type of filter for ≥11 days or a maximum of 42 closed or 20 drainable bags.

The primary endpoint (difference in frequency of ballooning between the full-circle filter and the dual filter) was analyzed using a Poisson regression model for the number of events with filter type and test period as fixed effects, and participant as a random effect. Participant was included as a factor because of the crossover design, and test period was used to adjust for a possible trend or learning effect. Ostomy solution wear-time (calculated using the time of ostomy solution change) was accounted for as an off-set in the analysis of the primary endpoint. Mean (standard deviation [SD]) values were reported for the number of ballooning events per person and ballooning events per person per day.

The difference in time (h) to ballooning was a survival endpoint; values were censored when ballooning did not occur during the wear-time of an ostomy solution. Difference in time to ballooning was analyzed using an accelerated failure time model with filter type, test period, and participant as factors. Hazard ratios (HRs) were determined together with 95% confidence intervals (CI). The frequency of pancaking and the frequency of odor with the full-circle filter versus the dual filter were analyzed with the same Poisson regression model used for the primary endpoint. Ostomy solution discretion was tested using an ordinal logistic regression model with filter type as an explanatory variable and participant as repeated measurement. The null hypothesis assumed no difference in the multinomial distributions defined by the two types of filter. Mean (standard error of the mean [SEM]) wear-time was determined using a mixed model with filter type and test period as fixed effects, and participant as a random effect. Safety outcomes (AEs and ADEs) were summarized according to filter type using descriptive statistics.

For all comparisons, *p* < 0.05 was considered statistically significant. All analyses were conducted using SAS software version 9.2 (SAS Institute Inc., Cary, NC, USA).

## 3. Results

### 3.1. Participant Disposition, Demographics and Clinical Characteristics

**Figure 3:**
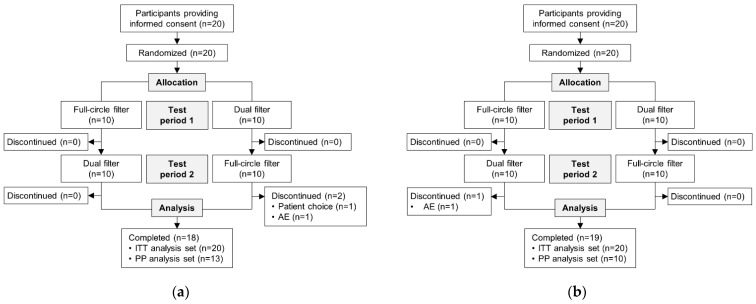
Participant disposition for (**a**) participants with a colostomy, and (**b**) participants with an ileostomy. AE = adverse event; ITT = intention-to-treat; PP = per-protocol.

Overall, 20 people with a colostomy were randomized in CP210OC; 18 (90.0%) participants completed the trial (Figure 3a). A total of 20 people with an ileostomy were randomized in CP211OC (Figure 3b); 19 (95.0%) participants completed the trial.

Baseline demographics and clinical characteristics are presented in Table 1. At baseline, 21.1% (n = 4) of participants with a colostomy and 52.6% (n = 10) of participants with an ileostomy experienced ballooning every day. Among participants with a colostomy, ballooning was most common during the day (55.6%, n = 10), whereas ballooning was most common at night (73.7%, n = 14) among those with an ileostomy.

### 3.2. Ballooning

**Figure 4:**
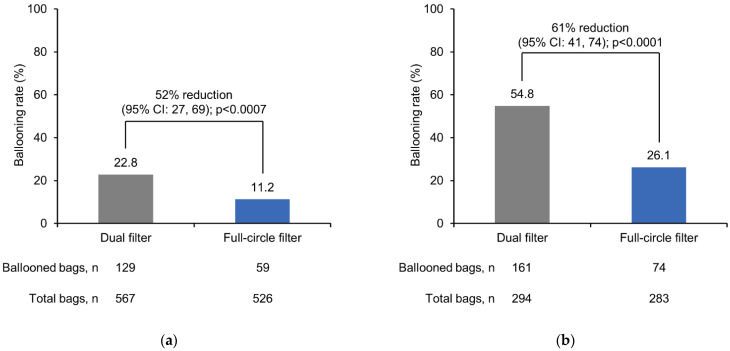
Frequency of ballooning with the full-circle filter versus the dual filter for (**a**) participants with a colostomy (n = 20), and (**b**) participants with an ileostomy (n = 20). Data are presented for the ITT analysis set. CI = confidence interval; ITT = intention-to-treat.

The frequency of ballooning was significantly lower with the full-circle filter compared with the dual filter in participants with a colostomy (*p* < 0.0007) and in participants with an ileostomy (*p* < 0.0001) (Figure 4).

For the colostomy and ileostomy groups, the number of ballooning events per person was lower with the full-circle filter versus the dual filter (Table 2). This trend remained the same when adjusted by the number of test days (Table 2). The percentage of participants who experienced less than five ballooning events was higher with the full-circle filter than with the dual filter among people with a colostomy (85.0% versus 50.0%) and people with an ileostomy (65.0% versus 30.0%). On an individual level, most participants experienced a lower frequency of ballooning (events per bag) with the full-circle filter versus the dual filter: 11 (55.0%) participants with a colostomy and 13 (65.0%) participants with an ileostomy. Seven people with a colostomy and four people with an ileostomy reported very few ballooning events in both test periods. Removing these outliers did not alter the findings of the primary endpoint.

**Figure 5:**
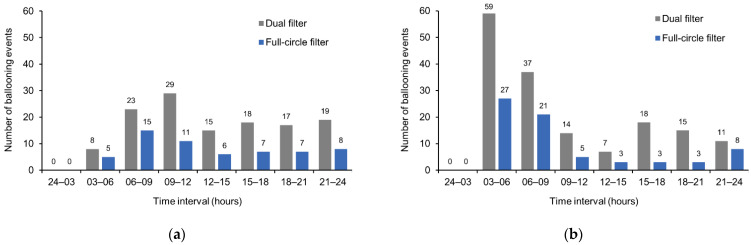
Distribution of ballooning events over 24 h with the full-circle filter and the dual filter in (**a**) participants with a colostomy (n = 20), and (**b**) participants with an ileostomy (n = 20). Data are presented for the ITT analysis set. Time intervals are indicated using the 24 h clock. ITT = intention-to-treat.

Considering the timing of ballooning events, for all time intervals and in participants with a colostomy or an ileostomy, the incidence of ballooning was lower with the full-circle filter versus the dual filter (Figure 5). No events were registered in the 24–03 h time interval.

The time to ballooning was significantly longer with the full-circle filter compared with the dual filter in participants with a colostomy (HR: 0.487; *p* = 0.0001) and in participants with an ileostomy (HR: 0.356; *p* < 0.0001). Consequently, with the full-circle filter versus the dual filter, the time to ballooning was 74% (95% CI: 35, 124) longer for colostomy bags and 82% (95% CI: 60, 106) longer for ileostomy bags.

### 3.3. Pancaking, Odor Problems, and Discretion

There were no statistically significant differences in the frequency of pancaking and odor problems with the full-circle filter versus the dual filter in participants with a colostomy or an ileostomy (Table 3). Perception of ostomy solution discretion was similar with the two filters, regardless of stoma type.

### 3.4. Ostomy Solution Wear-Time

Among participants with a colostomy, there were no significant differences in mean (SEM) ostomy solution wear-time with the full-circle filter (10.6 [0.19] h) versus the dual filter (10.3 [0.19] h). However, among those with an ileostomy, ostomy solutions with the full-circle filter were, on average, worn for 3.3 h longer (20.5 [0.44] h) than ostomy solutions with the dual filter (17.3 [0.40] h) (*p* < 0.0001).

### 3.5. Sensitivity Analyses

The results of the sensitivity analyses performed for the primary endpoint (frequency of ballooning) in the PP analysis sets supported the findings of the ITT analyses. For both analysis sets, the test period (1 or 2) had no effect on the primary endpoint (*p* > 0.05 for the ITT and PP sets).

### 3.6. Safety

Overall, six AEs were observed in four (20.0%) participants with a colostomy; of which, one was considered an ADE (with the full-circle filter). A total of five AEs were observed in five (25.0%) participants with an ileostomy; of which, three were considered ADEs (all with the dual filter). One AE (an abscess), in a participant with an ileostomy, was serious but not considered related to the trial intervention (ostomy solution with the dual filter). All other AEs/ADEs were not serious.

In each trial, one participant discontinued due to an ADE. One participant with a colostomy experienced skin redness and pruritus of moderate severity under the baseplate of the ostomy solution (ostomy solution with the full-circle filter); this was considered device-related but not related to the full-circle filter itself. The participant was referred to the care of their own doctor and stoma nurse, and the ADE resolved within 3 days. One participant with an ileostomy experienced skin redness above the stoma of moderate severity (ostomy solution with the dual filter); this was considered device-related, probably due to bag leakage, and resolved within 2–4 days.

## 4. Discussion

In recent years, considerable advances have been made in the design and function of ostomy solutions, including the addition of a filter [1,4], and a pre-filter. However, issues related to filter performance—such as ballooning, pancaking, leakage, and odor—remain highly prevalent among people living with a stoma [9,10,11,12]. Despite the high prevalence of filter-related issues, published research evaluating the performance of stoma bag filters is limited. The results for the two 4-week, open-label, randomized, two-way crossover trials presented here add to the knowledge in this area and help address the data gap.

These two trials show that the frequency of ballooning was significantly lower with the full-circle filter compared with the dual filter in participants with a colostomy and in participants with an ileostomy. It could be hypothesized that a reduction in ballooning frequency may reduce the need for frequent ostomy solution changes and, therefore, extend product wear-time. Indeed, time to ballooning was significantly longer with the full-circle filter versus the dual filter for participants with a colostomy (*p* = 0.0001) and participants with an ileostomy (*p* < 0.0001). However, differences in wear-time between the two filters were not consistent across the two trials. For participants with a colostomy, wear-time was not significantly different between the two filters, whereas, among participants with an ileostomy, wear-time was significantly longer with the full-circle filter versus the dual filter (*p* < 0.0001). This finding is, perhaps, unsurprising. Participants with ileostomies were provided with drainable bags, which could be emptied and, therefore, could be worn for a longer period of time than the closed bags used by participants with colostomies. Considering that participants were instructed to change their ostomy solution whenever ballooning occurred, a reduction in ballooning may extend wear-time to a greater extent for those with ileostomies than those with colostomies, who must change their ostomy solution frequently, irrespective of ballooning.

The occurrence of ballooning events at night can result in sleep disruption [9]. One descriptive study in individuals with an intestinal stoma reported poor sleep quality in 66.2% of participants [15]. The authors noted that very few people were using stoma bag filters and recommended their addition [15]. Sleep was also significantly poorer in people with an ileostomy than in those with a colostomy (*p* < 0.05), which the authors suggested could be due to leakage anxiety and the need to empty the bag [15]. In the present trials, at baseline, ballooning was more common at night in participants with an ileostomy (73.7%) and more common during the day in those with a colostomy (55.6%). In both groups, use of the full-circle filter reduced the number of ballooning events at night compared with the dual filter, which could, potentially, improve sleep.

For the secondary endpoints, frequency of pancaking and odor problems, and ostomy solution discretion, no significant differences were observed between the full-circle filter and the dual filter. Like ballooning, pancaking is a common yet under-researched filter-related issue among people living with a stoma [11]. A key challenge in the design of a stoma bag filter is achieving a balance between gas removal (to avoid ballooning) and retention (to avoid pancaking). Minimization of ballooning issues was prioritized during the development of the full-circle filter system, with a large pre-filter specifically included to prevent the carbon filter from becoming clogged with stomal effluent. In light of this targeted development strategy, the full-circle filter may be expected to have a minimal effect on the occurrence of pancaking. It is important to highlight that the frequency of pancaking prior to enrollment was not recorded in the present trials and, therefore, it is difficult to fully assess the effect of the full-circle filter and the dual filter on the occurrence of pancaking issues. Nevertheless, it is encouraging that the frequency of pancaking was not significantly different with the full-circle filter versus the dual filter, suggesting that the altered design of the full-circle filter is unlikely to worsen pancaking issues compared with the original dual filter.

In addition to bag-related issues, ostomy solution discretion is highly valued by people living with a stoma [16,17]. Many individuals are concerned about the appearance of their ostomy solution [16,17], with one in three worrying that the bag may be visible through their clothing or that its odor would be noticeable [9]. Therefore, ostomy solution discretion and odor control were important considerations. The finding that the full-circle filter was not perceived as significantly less discrete than the dual filter or associated with a higher frequency of odor problems, may provide welcome reassurance for people living with a stoma.

A major strength of this research is the randomized crossover trial design. A crossover design was chosen as fewer participants would be required to power the primary endpoint compared with a parallel-group study. Furthermore, a crossover design was a suitable choice considering the short-term effect of the interventions and the stable condition of the participants [18]. The randomization of participants together with the crossover design minimizes bias as participants serve as their own control. Additionally, the two trials were statistically powered for the primary endpoint.

A limitation of the research is the delayed publication of these clinical trial data. Following the positive outcomes of the trials, the commercial introduction of the full-circle filter was prioritized. Indeed, at the time of trial completion, the value of publication was not fully recognized. However, considering the lack of published data regarding stoma bag filters, the persistent high prevalence of filter-related issues [9,12], and the fact that people living with a stoma consider ostomy solution performance to be a research priority [13], it is important to publish these data, regardless of the delay. Another limitation of the present trials is their open-label design. Blinding is frequently used in clinical trials to determine the ‘true effect’ of an intervention without the influence of preconceptions from participants and investigators [19]. However, blinding is inherently challenging across trials of medical devices, which is acknowledged by authorities. In the present trials, visible differences in the appearance of the full-circle filter and the dual filter meant that successful blinding of trial participants was not feasible. Indeed, limited opportunities for blinding is a trade-off for attempting research in the field of ostomy product solutions. Lastly, for inclusion in the trials, participants needed to experience at least one ballooning event per week. Therefore, it is unclear what effect the full-circle filter would have in a broader population. Large-scale, observational studies are needed to gather additional information on filter performance in a real-world setting.

In conclusion, the results of these two randomized crossover trials show that use of the full-circle filter significantly reduced the frequency of ballooning events versus the dual filter in individuals with a colostomy or an ileostomy. Considering the lack of published research relating to stoma bag filter performance, these data provide valuable information for health care practitioners who support people living with a stoma.

## Figures and Tables

**Table 1:** Baseline demographics and clinical characteristics.

	Colostomy (n = 20)	Ileostomy (n = 20)
**Demographics**		
Sex	(n = 19) ^a^	(n = 20)
Male	11 (57.9)	13 (65.0)
Age, years		
Mean (SD)	66 (6)	60 (11)
Range	53–75	40–78
**Clinical characteristics**		
Size of stoma bag used	(n = 18) ^b^	(n = 20)
Mini	1 (5.6)	1 (5.0)
Midi	15 (83.3)	16 (80.0)
Maxi	2 (11.1)	3 (15.0)
Frequency of ostomy solution change	(n = 19) ^a^	(n = 19) ^a^
1 every other day	1 (5.3)	3 (15.8)
1 per day	5 (26.3)	15 (78.9)
2 per day	7 (36.8)	1 (5.3)
3 per day	3 (15.8)	0 (0.0)
>3 per day	3 (15.8)	0 (0.0)
Frequency of ballooning	(n = 19) ^a^	(n = 19) ^a^
1 time per week	0 (0.0)	1 (5.3)
>1 time per week	15 (78.9)	8 (42.1)
Every day	4 (21.1)	10 (52.6)
Time of ballooning	(n = 18) ^c^	(n = 19) ^a^
Night	3 (16.7)	14 (73.7)
Day	10 (55.6)	1 (5.3)
Night and day	5 (27.8)	4 (21.1)

^a^ Baseline data are missing for one participant; ^b^ one participant used more than one bag size, and one participant did not report bag size; ^c^ baseline data are missing for two participants. Data are presented for the ITT analysis set as n (%), unless otherwise specified. ITT = intention-to-treat; SD = standard deviation.

**Table 2:** Number of ballooning events per person with the full-circle filter versus the dual filter.

	Colostomy (n = 20)	Ileostomy (n = 20)
**Ballooning events per person**		
Full-circle filter	3.0 (3.71)	3.7 (3.29)
Dual filter	6.5 (6.54)	8.1 (6.02)
**Ballooning events per person per day**		
Full-circle filter	0.3 (0.33)	0.3 (0.36)
Dual filter	0.7 (1.22)	0.9 (0.87)

Data are presented for the ITT analysis set as mean (SD). ITT = intention-to-treat; SD = standard deviation.

**Table 3:** Frequency of pancaking or odor problems with the full-circle filter versus the dual filter (number of bags with pancaking or odor problems/total number of bags).

	Colostomy (n = 20)	Ileostomy (n = 20)
**Frequency of pancaking**		
Full-circle filter	13.7 (72/526)	7.1 (20/283)
Dual filter	9.2 (52/567)	5.4 (16/294)
*p*-value	0.3289	0.7846
**Frequency of odor problems**		
Full-circle filter	11.2 (59/526)	2.8 (8/283)
Dual filter	12.3 (70/567)	3.1 (9/294)
*p*-value	0.8583	0.7586

Data are presented for the ITT analysis set as % (n/N). *p*-values were calculated for the full-circle filter versus the dual filter. ITT = intention-to-treat.

## Data Availability

Data presented in this publication are available on the relevant ClinicalTrials.gov pages for CP210OC (NCT01273038) and CP211OC (NCT01272869).
